# Traditional Arabic & Islamic Medicine (TAIM): Principles of Dietary Practices and the TAIM Food Pyramid

**DOI:** 10.5539/gjhs.v17n2p36

**Published:** 2025-03-30

**Authors:** Sara N. AlRawi, Suzanna M. Zick

**Affiliations:** 1 Independent Researcher, Ann Arbor, U.S.; 2 Department of Family Medicine, University of Michigan, Ann Arbor, U.S.

**Keywords:** Cultural competency, Food pyramid, Middle Eastern Diet, Diet Therapy, Traditional Arabic and Islamic medicine (TAIM)

## Abstract

Middle Eastern and Mediterranean culinary tradition and dietary practices are used interchangeably due to geographical proximity and similarities in their cuisine. While these regions share historical impact and cultural exchanges there are differences that delineate each region’s dietary influences. Dietary practices are one of five core elements of an overarching Traditional Arabic and Islamic Medicine (TAIM) conceptual framework, yet current dietary guidelines are lacking. We introduce the TAIM food pyramid as a visual representation of TAIM’s historical and regional nuance, illustrate dietary patterns, and provide a cultural competency strategy. We used Oldways as a model for creating the TAIM food pyramid while illustrating its extension of a wholistic paradigm representing the medical and indigenous traditions, beliefs and practices of a geographic region and cultural community. We focus on two important aspects: historical roots of traditional Arabic Medico-Culinary cuisine coupled with Islamic medicine and influences of Prophetic tradition on foods. The TAIM food pyramid is an illustration of a healing tradition emphasizing sound nutrition, food safety, social connection, community and sustainability. The pyramid is similar in structure to global traditional healing diets within the context of culture and lifestyle of the Middle East and North African communities. Despite trends in research focusing on the Mediterranean diet, little is known about the Middle Eastern diet. The absence of a TAIM food pyramid is a clear gap in research and a missed opportunity for further analysis. Health care delivery that is culturally competent improves health equity and translates to patient centered, whole-person care.

## 1. Introduction

Traditional Arabic medicine evolved to integrate elements of Islamic medicine embodied in the scripture of the Quran and prophetic tradition, yielding a comprehensive system of medicine defined as Traditional Arabic & Islamic Medicine, or TAIM (Alrawi & Fetters, 2012). TAIM emphasizes the prevention of disease and preservation of health with treatment based on diet being a key medical intervention within this framework (Alrawi & Fetters, 2012). Traditional Arab and Islamic physicians like Avicenna, believed patients should be treated first with diet and linked the natural effects of diet, lifestyle, emotions and the environment with disease and health restoration (Choopani & Emtiazy, 2015; M. A. Khan et al., 2015; Saad, 2015). Rhazes, supported the notion that diet plays a role in human physiology, pathology, health maintenance and treatment of disease (Nikaein et al., 2012). He is credited with saying ‘as long as you can heal with food, do not heal with medication (Zargaran et al., 2014). This innovative contribution led to the advancement of a medico-culinary tradition nourishing the body, mind and spirit which continues to thrive today in countries and traditions rooted in the Islamic world.

Middle Eastern and Mediterranean culinary tradition and dietary practices are often used interchangeably due to geographical proximity or similarities in their cuisine. While these regions share historical impact and cultural exchanges there are several differences that delineate each region’s dietary influences (Naja et al., 2015). Mediterranean cuisine typically includes those countries that border the Mediterranean Sea such as Greece, Italy, Turkey and some Arab nations in north Africa such as Morocco, Algeria and Tunisia (Turmo, 2012). This designation is problematic as it excludes countries such as Iraq, Yemen, Jordan, United Arab Emirates, Saudi Arabia, Oman, Kuwait, Bahrain and Qatar. Therefore, identifying Middle Eastern cuisine as Mediterranean is lacking depth and accuracy.

The population of the Arab world is approximately 500 million people (*World Bank Open Data: Free and open access to global development data*), with 3.7 million Americans having Middle Eastern or North African ancestry, though this number is likely greater due to the misrepresentation as racially “White” on the U.S. Census (*Arab American Institute*). Despite their considerable ethnic composition, clinical tools to guide TAIM dietary practices in this community are scarce at best. Culturally sensitive tools are therefore necessary to bridge access to care and to provide intercultural competence. A recognizable nutritional tool is the food guide pyramid (Marcus, 2013). In this manuscript, the authors introduce the TAIM food pyramid as a tool to highlight cultural diversity in nutrition and help provide intercultural competent dietetics education. We also present the framework behind the TAIM food pyramid.

## 2. Methods

We used Oldways (*Oldways*) as a model for creating a TAIM food pyramid. Oldways creates visual roadmaps to produce heritage diet pyramids. Similarly to the Oldways paradigm, the TAIM food pyramid celebrates the diversity and fusion of culinary techniques and practices of the Middle East and North Africa, yielding a tapestry of traditional Arabic dietary practices with Islamic medicine influences. In addition, the TAIM food pyramid’s dietary principles are an integral component of an overarching conceptual model and therefore an essential element of TAIM’s integrative medical system (Alrawi & Fetters, 2012). The TAIM food pyramid is an extension of a wholistic model representing the medical and indigenous traditions, beliefs and practices of a geographic region and cultural community and thereby imparting the rituals and lifestyle with which people identify and live (Alrawi & Fetters, 2012). To illustrate this, we focus on the historical roots of traditional Arabic Medico-Culinary cuisine on food culture in the Islamic world.

### 2.1 Historical Evolution of Traditional Arabic Medico-Culinary Practice

The earliest agronomical work in Arabic on Nabatean agriculture, attributed to Ibn Wahshiyyah contains medical and culinary uses for nearly 360 plants (Waines, 1999). Evidence of culinary manuals and treatises written by Rhazes include the ‘Book on the benefits of food and avoidance of their harmful effects’, where he explained the importance of food and diet essential for treatment of disease, prevention and health preservation (Nikaein et al., 2012; Zargaran et al., 2014). Rhazes introduced many types of food for medicinal use together with details of their classifications, methods of preparation, physical properties and therapeutic modes of action (Ansari et al., 2018). Other contributions include a culinary manual compilation by Ibn Sayyar al-Warraq which includes advice on the benefits of certain recipes to the body and avoidance of the harmful effects of such dishes (Nasrallah, 2007). A 14th century Egyptian manuscript translated as ‘Treasure Trove of Benefits and Variety at the Table’ details over 800 recipes for dishes, digestives and beverages (Nasrallah, 2017). In his medical work, Ibn al-Qayyim introduced the effect of diet on both health and illness as potentially more effective than that of drugs (Deuraseh, 2014).

Physicians of the Arab and Muslim world also drew upon Hippocrates’ humoral theory, Galen’s classification of foods and medications and advanced this knowledge based on their own observations into a sophisticated system of medicine (Ahmad, 2023; Hajar, 2021). According to humoral theory, each body organ has a special temperament, or mizaj (Moradi et al., 2013). Temperament relates to the blending and interaction of four elements: fire, air, water and earth (Miraj, Alesaeidi, Kiani, et al., 2016). These elements are considered the base of all living entities including plants, animals and minerals (M. A. Khan et al., 2015). When combined, these elements produce humors. Balance between the humors indicates health, whereas disease occurs when there is an imbalance, e.g., excess or deficiency in the humor (Miraj, Alesaeidi, & Kiani, 2016). Balancing humors and restoring health is initially attempted by means of proper nutrition, namely by means of foods consistent with the patient’s temperament (Moradi et al., 2013). Improper eating is therefore a primary culprit in dystemperament and diet therapy as a means to maintaining homeostasis (Kopaei et al., 2016). The order in which food should be served and consumed is also important to optimize digestion and foster humoral balance. Herbs and spices were considered for their medicinal properties thereby contributing to the overall therapeutic effect of the diet (Petrovska, 2012).

The medical tradition of diet gradually became a culinary practice. An analysis of medico-culinary sources reveals that there was a cuisine specifically developed for purposes of healing (Waines, 1999). Recipes were formulated with indications for health restoration and included references to the humoral qualities of the various ingredients, and to the quantity and the moment in the meal when they were to be consumed to ensure a physiological effect. Hospitals were built to grow common medicinal foods and herbs (Pitchon, 2016). Moreover, the pharmacy prepared drinks, syrups and medicines, comprising a variety of fine electuaries and high quality preserved medicinal fruit drinks (Chipman, 2007) and added to a base consisting of citrus fruits, juice, myrobalan (*Amlaj)* or Indian gooseberry, but also flower petals, or even roots such as ginger, parsnip and carrots (Pitchon, 2016). Similarly, in domestic households the culinary manuals indicate preparations for several kinds of vegetable concoctions, electuaries, stomachics (a medicine or tonic that promotes the appetite or assists digestion), medicinal powders and fruit juices (Pitchon, 2016).

### 2.2 Influence of Islamic Medicine & Prophetic Tradition on TAIM Culinary Practices

Health within the Islamic framework is described as a state of complete physical, psychological, social and spiritual wellbeing (Rassool & Sange, 2014). Health behavior is amongst the most fundamental components of Islamic practice including discipline in eating, dietary habits, temperance and abstinence that play a major role in augmenting the principles of traditional Arabic medicine (Jabbaripour et al., 2020). Rooted in Islamic teachings is the concept of sharing meals with others and promoting charity, whereby Muslims feed the hungry and the vulnerable thereby fostering community. According to tradition, a man asked the Prophet, “What Islamic traits are the best?” The Prophet said, “Feed the people, and greet those whom you know and those whom you do not know” (1996b). The Prophet also said, “No Muslim plants a tree or sows a seed and then a bird, or a human, or an animal eats from it but that it is charity for him” (Al-Bukhari & Al-Bukhari, 1996a) and “he is not a believer whose stomach is filled while his neighbor goes hungry” (Al-Bayhaqi, 1990). Lastly, the practice of charity has a direct correlation to the role of an individual’s recovery from illness and restoration of health (Koenig & Shohaib, 2014).

Islam stressed the importance of moderation in its role in providing balance to the body (Syed Mohideen et al., 2022). Ibn Qayyim Al-Jauziyah, in his writing on ‘Healing with the Medicine of the Prophet’ (Al-Jauziyah, 1999), sheds light on how to observe a balanced intake of food and drink as both deficiency and excess hinder bodily function thereby causing disease. Islamic sages advised mindfulness in eating, enjoying meals in a peaceful place without haste or preoccupation with another task (Koenig & Shohaib, 2014). Emphasis was placed on seasonal, nutritious and sustainable food principles. For this reason, Islam has prohibited some foods and beverages, such as alcohol, due to their ill effects (M. Khan et al., 2015). Though Arabs are credited with discovering how to distill alcohol, also known as Arak, it is enjoyed by minority populations due to Islamic dietary laws (Matthee, 2014). Some regard Arak as possessing healing properties as it is considered a digestive aid and contains antimicrobial properties. Arak has an aniseed base and differentiates itself through regional attributes to include dates, sugar, plums, figs, and molasses (Cabaroglu et al., 2023).

It’s evident that dietetics became a central element in the system of healthcare operating at this time. Ibn Habib, an Andalusian polymath, drew upon two distinct sources, the Traditions of the Prophet and the Greek humoral theory (Waines, 1999). An extract from “The physician’s dinner party” written by the eleventh-century doctor, Ibn Butlān, clearly establishes the priority of dietetics over other forms of medical care in the Islamic tradition whereby he states, “reject treatments by drug whenever it is possible to treat by food” (Pitchon, 2016). The Qur’an and prophetic tradition guide various aspects of daily life, including the use of household remedies and medicinal herbs such as the black seed and its oil. Food is believed to have an effect on the body and soul and therefore the notion of spiritual nutrition is revered (Cabaroglu et al., 2023). Spiritual practices such as fasting is common amongst Muslims, Christians and Jews. Ibn Sina and Ibn Qayyim believed fasting to be a method of food reduction and one of the best ways to stay healthy (Nozad et al., 2016). During the ritual fast whereby, Muslims abstain from eating and drinking, they also partake in feeding a vulnerable person, being absorbed in the remembrance of God, fostering patience, refraining from indulgence, showing humility, and purifying themselves (Ahmad & Goel, 2012). While the tradition around fasting varies, the concept of fasting as a physical and spiritual remedy is shared by Arab Christians who observe fasting during Lent and Jews who observe fasting during Yom Kippur (Akram, 2016).

## 3. Results

**Figure 1.**
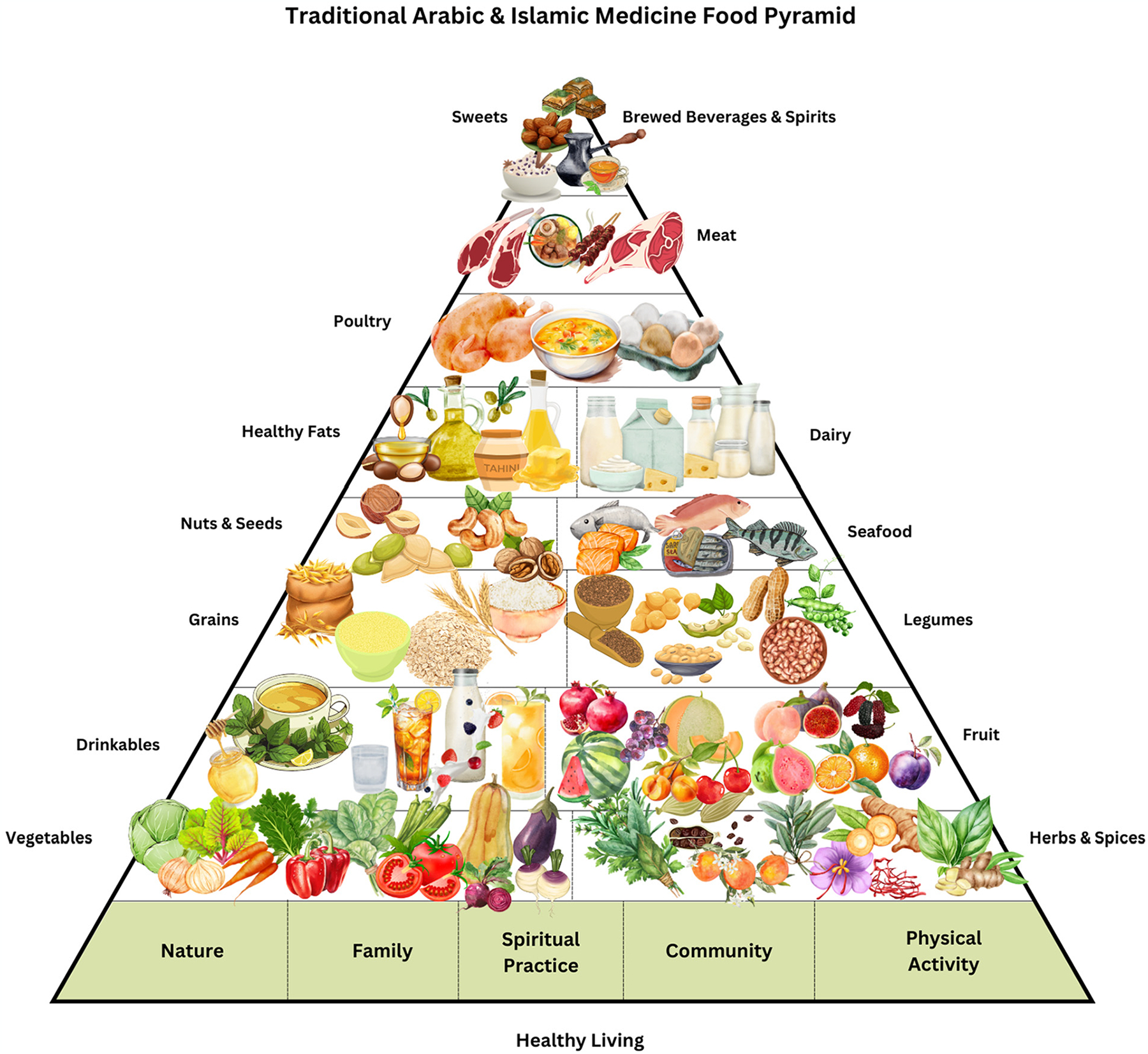
Traditional Arabic & Islamic Medicine Food Pyramid

The TAIM food pyramid is shown in Figure 1. Although culturally and regionally unique, the TAIM food pyramid is comparable to other traditional diets which center around seasonal and local ingredients with a foundation of vegetables, fruit, whole grains, pulses, herbs and spices, healthy fats, poultry, meat, seafood and traditionally produced dairy products. At the base of the TAIM food pyramid, lifestyle components stemming from people of the Middle East and North Africa’s food traditions and culture such as physical movement, encouraging community, connectedness to nature, mindfulness in eating, spiritual practices, and a healthy way of living.

In the TAIM pyramid, we have identified 8 additional tiers. Table 1 is a descriptive of each tier, highlighting key elements of cultural heritage as well as seasonal and regional produce.

The closer to the base of the pyramid, the larger the tier in terms of visually representing the desired percentage of food consumption whereas closer to the top of the pyramid are foods representing a smaller percentage of the diet and may be reserved for ceremonies, holidays or special occasions.

The TAIM food pyramid is a holistic approach that considers the interactions between food, culture and nutrition. Similarly to the Mediterranean diet, the TAIM food pyramid identifies patterns that are rich in plant-based foods, possess socio-cultural values, boast health benefits, emphasize local and seasonal foods and follow a traditional eating pattern revolving around relationships, culture and wellbeing. These enduring traditions make the TAIM food pyramid inherently sustainable.

## 4. Discussion

In this paper, the authors propose a TAIM food pyramid for application within the academic, medical and public health sphere. Knowledge of TAIM’s medical and culinary traditions in a cohesive framework is lacking, yet these practices have consequences for health behavior and education. This pyramid is theory driven and grounded in an overarching conceptual model (Alrawi & Fetters, 2012). Historically, a pyramid represented advice on food consumption (Raikar, 2024), only recently has this been modified to include elements of lifestyle such as movement. The TAIM food pyramid is a depiction of a rich therapeutic and culinary healing tradition emphasizing sound nutrition, food safety, social connection, community and sustainability. These enduring traditions continue to be passed down through generations as these practices symbolize a group’s culture and are therefore at the core of a patient’s identity and societal connections (Mingay et al., 2021).

The authors have demonstrated that Middle Eastern and Mediterranean dietary patterns vary, and the interchangeable use of the guidelines must be reconsidered. Adherence to the Mediterranean diet can be a barrier due to cultural beliefs or norms. A study by Benhammou, S. et al. (2016) looked at a comparison of Mediterranean diet compliance between European and non-European populations in the Mediterranean basin and found that the Spanish diet was shown to be closer to the Mediterranean diet than that of Morocco or Palestine (Benhammou et al., 2016). In a systematic literature review of 50 studies pertained mostly to the European Mediterranean countries, with fewer studies from the Middle Eastern and North African Mediterranean countries, found that the vast majority reported low or moderate adherence to the Mediterranean Diet (Obeid et al., 2022). Najah, F. et al. (2015) proposed a Middle Eastern version of the Mediterranean Diet when ascertaining adherence among Lebanese adults, thus necessitating a culturally appropriate blueprint of dietary practices (Naja et al., 2015). While there are similarities between the countries along the Mediterranean, there are also distinguishing differences in their respective food habits. Interestingly, food habits of neighboring countries are shown to be closer than those on opposite sides of the Mediterranean Sea, leading one to question large scale applicability of the Mediterranean diet (Noah & Truswell, 2001).

Healthcare system implications exist for understanding the role of dietary practices among minority populations. Racial and ethnic minorities have higher morbidity and mortality from chronic diseases necessitating more interactions with the healthcare system (National Academies of Sciences Engineering and Medicine; Health and Medicine Division et al., 2017). When there is a disconnect among providers, organizations and health care systems in providing culturally competent care, patients face a higher risk of experiencing negative health consequences, receiving poor quality care or being dissatisfied with their care. As healthcare providers strive to deliver multicultural orientation and culturally sensitive care, ensuring inclusive and equitable patient care is imperative (Williams et al., 2016). The TAIM food pyramid is an exquisite example of a culturally competent strategy, as it conveys a powerful symbol of cultural identity.

The preservation of TAIM’s rich medical tradition and the positive role it plays in the food culture is necessary. While a food pyramid does not capture a large geographic area, it does render a characterization of social and cultural considerations. Routine evaluation of the pyramid, assessing adherence, refining its use among both patients and providers is vital through the exploration of the long-term efficacy of nutrition-related health promotion efforts. It’s unclear whether the role of dietary practices in TAIM include the implementation of healing foods in a culinary fashion (Alvarez Millan, 2010). More research is needed to decipher whether diet is a self-care practice or whether practitioners are providing prescriptions for specific foods. There is a rich oral tradition which may mean variation in the way a recipe is prepared. Regional differences contribute to the use of certain herbs, spices and access to certain foods thereby impacting the desirable effect. Limited access to practitioners knowledgeable in TAIM may result in professional oversight of such therapies (Saab, 2015). Tradition, social influences, religion and health motivations continue to drive the complexities influencing food behavior (Mingay et al., 2021). A relevant paradigm is necessary to improve communication and drive research around beliefs and the value of food culture as a way of life in the American Arab and Muslim community (Mingay et al., 2021).

## 5. Conclusion

Traditional Arabic & Islamic Medicine (TAIM) views health as a state of balance between the body, mind and spirit, it is value based and non-reductionistic and both holistic and preventive in its approach to lifestyle and wellness. We can say TAIM is a humanistic form of medicine, an interdisciplinary approach emphasizing health for the individual and the community. This extension of community has always been an integral aspect of the Islamic faith and certainly as it pertains to health whereby the health of communities reflects the health of their individual members.

Diet therapy is a core element of the TAIM conceptual model and fundamental to healthcare seeking behavior. Traditional Arabic & Islamic medicine contains a rich medical and culinary tradition around sustainable and healing foods based on the principle of balancing bodily humors within a framework of dietary laws and practices whereby recommendations are tailored to the individual’s needs. Despite trends in research focusing on the Mediterranean diet, little is known about the Middle Eastern diet albeit at times these categories are being used interchangeably. It is not our goal to debate the merits of one diet versus another, rather to encourage a food-based approach to healthy eating rooted in traditional diets. We introduce the TAIM food pyramid as an exploration into this dynamic paradigm, a representation of Middle Eastern and North African food culture, medical and culinary tradition and a sophisticated framework for health and healing.

The absence of a TAIM food pyramid is a clear gap in research and a missed opportunity for further analysis. There are research applications for the study of TAIM dietary practices on health outcomes including intervention studies. It is our hope that the TAIM food pyramid will be implemented as a culturally competent strategy, promoting healthy eating traditions and stressing the need for successful dietary interventions. The interplay between culture and health for the diverse American Arab and Muslim patient population which recognizes traditions leads to improved adherence. The integration of the TAIM food pyramid into contemporary public health nutrition strategies is a practical approach to the changing landscape of nutrition delivery. With the rise in chronic disease, more patients are looking to traditional systems of health and value-based healthcare. For many, this is an extension of their belief system and worldview. Health care delivery that is culturally competent addresses health disparities and improves health equity, and translates to patient centered, whole-person care.

## Figures and Tables

**Table 1.** Common Traditions, Foods and Flavors of the Middle East and North African Diet

Tier	Groups	Notes
I	Vegetables	Freshly sourced vegetables in raw, cooked, dried or pickled form including leafy vegetables such as dandelion greens, jute leaves, cabbage, cress, lettuce, spinach, chard; root vegetables such as onions, carrots, turnips, radish and beets as well as eggplant, okra, squash, potato, tomato (based on common use), cucumber and pepper varieties. Wild edible plants regarded as valuable within rural areas have at times been necessitated by famine but have a rich heritage and are commonly used by local communities for their health and medicinal qualities. Wild edible plants include grasses, fungi and shrubs.
	Herbs & Spices	Medicinal and culinary herbs and spices such as cumin, coriander, cardamom, harissa, caraway, za’atar, turmeric, anise, sumac, mint, sage, cinnamon, basil, ginger, garlic, clove, rosemary, nutmeg, fenugreek, dill, fennel, parsley, black and Aleppo pepper, saffron, dried rose petal and lavender are commonly used. Wild edible herbs include purslane and wild calla lily.
II	Fruits	Seasonal fruit in raw, dried, preserved or juice form including figs, pomegranates, dates, apple, citrus, apricot mulberries, persimmons, quince, melons, grapes, plums, guava, peaches, cherries, olives and green almonds. Fruit molasses, particularly pomegranate, date and grape are used in preparation of salads, beverages, main courses and sweets.
	Drinkables	Tisanes including mint, cinnamon, rose hip, chamomile, lemon verbena, lemongrass, marjoram and sage. Honey water tea. Hydrosols including rose and orange blossom water. Juice including Jallab (made with date syrup, grape molasses or pomegranate syrup, rose water and garnished with nuts), Tamar hind (sweet tamarind), Karkade (dried hibiscus flower tea), Qamar al-Din (apricot juice), Sobia (starchy sweet coconut milk), Erk Sous (licorice root), pomegranate and sugar cane juice. Ayran (yogurt mint drink). Water. Some drinkables are often enjoyed during certain rituals, social gatherings and religious holidays.
III	Grains	Grains and legumes can be considered the basis of the diet offering nutritious plant-based protein. Native grains such as farro, wheat varieties including bulgar wheat, spelt, wheat berries, kamut, freekeh as well as rice oats, barley and couscous. Healthy ancient grains are consumed throughout the day as either a foundation for salads, soups and stews or cooked into a side dish seasoned and complemented with herbs, spices, dried fruit and nuts. Aside from whole grains, rice and bread are eaten at almost every meal.
	Legumes	Pulses are more than just ingredients in the Arab culinary world, their significance touches aspects of history, culture, and ecology. Legumes such as lentils, chickpea, green peas, peanuts as well as fava, white, lupine and lima beans.
IV	Seafood	Seafood is a popular protein option because it doesn’t require the same religious ritual slaughter as other animals. Seafood includes predominantly fish such as sardines, mackerel, emperor fish, barramundi, tilapia, Nile perch, snapper, grouper, sole and red mullet as well as some varieties of shellfish.
	Nuts & Seeds	Nuts and seeds display a significant role in the Middle Eastern diet as well as in cultural traditions and customs around hospitality and healing practices. Often used in raw, toasted, or ground into paste or sauce form and used as a snack, in cooking or baking. Common varieties include sesame, sunflower, watermelon, poppy and pumpkin seeds, chestnut, almonds, pistachios, walnuts, cashews, hazelnuts and pine nuts.
V	Healthy Fats	Olive oil cultivation originated in the Middle East and is therefore the most recognizable fat used. However, animal fats have also been an integral component of the culinary tradition of this region and include camel hump fat and layeh (tail fat, sheep). Clarified butter, or *samneh*, is made using herbs and spices then fermented for months or even years. Plant based fats include tahini, argan and black seed oil.
	Dairy	Fermented milk products, derived from various sources such as cow, sheep, goat, water buffalo, and camel milk, have a long history in the Middle East and hold cultural, medicinal, and economic significance. These foods are often flavored with herbs, spices and combined with grains for health promoting benefits. Varieties include fermented yogurt and kefir, thickened yogurt, buttermilk, kishk (dried yogurt), preserved yogurt, labneh (preserved cheese) and ashta (clotted cream).
VI	Poultry	A variety of poultry is used such as chicken, Cornish hens, quails and pigeons including organ meats as well as bone and meat broth. Eggs including chicken, duck and quail.
VII	Meat	Since both Muslims and Jews do not eat pork, religion has impacted the cuisine by making other meats a primary choice. Preserved, cooked, smoked and cured varieties of lamb, mutton, goat, beef, rabbit, deer and camel. Organ meats, bone and meat broth. Guidance on humane and ethical rearing of animals and slaughter, emphasizing the importance of treating animals with kindness and respect is venerated.
VIII	Sweets	Sweets are associated with special occasions, holidays and rituals. Ingredients that are abundant in the region, such as dates, nuts like pistachios and almonds and various fruit are deeply intertwined with the agricultural history, tradition and cultural identity. Common varieties include Baklava (layered pastry dessert with nuts), halawa (halva), kanafeh (shredded filo dough crust, a sweet cheese filling, topped with a rose water flavored simple syrup), basbousa (semolina cake), maamoul (date, walnut or pistachio filled butter cookies), atayef (pancakes stuffed with cheese or ground nut mixture), muhallebi (rice milk pudding), warbat bil ashta (crispy filo dough filled with creamy center) and halawet el jibn (sweet cheese rolls).
	Brewed Beverages & Spirits	A perfect accompaniment to sweets includes beverages like black tea and coffee varieties with added herbs and spices such as cardamom, saffron and cloves, reflecting the essence of hospitality. Coffee cultivation techniques vary as well as brewing and preparation methods creating a myriad of flavors. Arak is a traditional, aniseed flavored distilled spirit with licorice notes and differentiates itself through regional attributes to include dates, sugar, plums, figs, and molasses.

## Data Availability

The data that support the findings of this study are available on request.
